# Homeopathy to the Rescue

**Published:** 1905-09

**Authors:** 


### TEXAS MEDICAL JOURNAL.

AUSTIN, TEXAS.

A MONTHLY JOURNAL OF MEDICINE AND SURGERY.

EDITED AND PUBLISHED BY
F. E. DANIEL, M. D.
Mrs. F. E. DANIEL, Business Manager.

Published Monthly at Austin, Texas. Subscription price $1.00 a year in advance

### HOMEOPATHY TO THE RESCUE.

Lo, the conquering hero comes!

My readers will have learned from the Associated Press dispatches that a Dr. R. B. Leach, of St. Paul, Minn., is in New Orleans for the purpose of demonstrating [?] to the medical profession and the world that arsenic (arsenious acid), in 1-100th grain doses, will not only *cure* yellow fever, but will prevent it! He offers to let any number of Stegomyia mosquitoes bite him,—he having already immunized himself by his specific (?). But the Board of Health turned him down cold. While recognizing that he is a fit subject for the fool killer, they don't want his blood on their hands. They have not forgotten Kibby and his cot. Kibby had a cot made of a peculiar construction, and his treatment was douching the patient with ice water while reclining on the cot. He demonstrated that his remedy would kill, pretty quick, by dying on his cot. Nevertheless, the papers say that everybody in New Orleans is taking arsenic, and *mirabile dictu!* the fever is said to be on the decline! It were useless to point out the relation of cause to effect. Leach feeds New Orleans on arsenic, and, *presto,*—yellow fever folds up its tents like the Arabs,—and *that,* too, after the United States army and navy and the Louisiana State troops, and the Mississippi State troops and the Marine Hospital and United State Public Health Service had taken a whack at it,—aided by oodles of money, and inspectors and "experts" *galore;* and after nearly a war between Louisiana and Mississippi and nearly a duel between their respective Governors—who said to each other "you are another."

All of which adds brilliancy and renown to the all-conqueror Leach and the all-potent arsenious arsenic!

I say the all-conqueror Leach and the all-powerful arsenic. For it will be remembered that this is the same Dr. R. B. Leach, a homeopath, who formerly lived at Paris, Texas, and who, in 1892, announced the great discovery that arsenic will cure and prevent *cholera!* And cholera, not knowing, I suppose, that Leach was on guard, armed with so potent a specific as arsenic, dared to invade these shores. Leach to the rescue,—as in this instance,—and the cholera slunk away, cowed by the mere announcement that Leach had this thing up his sleeve. He offered his services to President Benjamin Harrison, and Surgeon General Hamilton of the Marine Hospital Service, but they were not accepted. The mere threat was sufficient.

I published Dr. Leach's letter to the President in October, 1892, and editorial comments on it. I reproduce the following from that editorial. It applies as well now as then. Now let the conqueror go to India and with his arsenic tackle the plague which is destroying a million people a year, and *squelch that,* also. It is up to him.

(*From the "Red Back," October, 1892.*)

Some writer has sagaciously observed that there never was a crisis in human affairs—an emergency to be met, but some genius was raised up to meet it, or words to that effect. The truth of this observation has been demonstrated repeatedly. History, ancient and modern, abounds with striking illustrations of its correctness. Had we not, in the late war, Lee on the one side and Grant on the other, and Jefferson Davis and Abraham Lincoln, to demonstrate the truth of the observation from the standpoint, respectively, of Yank. and Confederate? Can not Napoleon Bonaparte and even Cincinnatus be cited? So, even when another noted crisis called, was not *Carius Marius* on hand? The breach was closed,—the country saved,—but *Carius M.,* where was he?

Well, the cholera came. Arising in the east, like a rested lion, he shook his mane and "lit out,"—seeking whom he might devour. People fled panic-stricken at his approach, and there was none to stay his progress. Across the Atlantic, at a single step, he came; like a schoolboy skipping across the spring branch. He growled at our door, and science, palsied with fear, yet with "disinfectants" in hand, stood irresolute. Behold! away off in Texas the cry is heard; the crisis having called, the genius was forthcoming! Dr. R. B. Leach, a highly respectable citizen of Paris, Texas, a homeopathic physician, eminent in his class, and, compared to whom, in light of the following, Hahnemann was a chump, conceives an idea. No, it's an inspiration! It is brilliant,—thrilling! Hahnemann said, "the hair of the dog will cure the bite," and, behold, the homeopathic doctrine—*similia similibus curantur*—is born! This modern Hahnemann now advances the beautiful theory that the hair of the dog will prevent the bite, hush the bark, and choke off the dog! That "like" will not only cure "like," but will prevent it.

Unlike the Keeleys and the Amicks (sordid creatures), Dr. Leach hastens to "give it away" without money and without price, fondly anticipating the time when the world will ring with the echo of his name,—when unborn generations will rise up and call him blessed, and fortune and fame will be his inheritance by right. Does he not intimate as much in his letter to the President? He will be canonized, and embalmed in history his name will go sounding down the ages, hpyhenated with that of Jenner, and coupled with all that is great and glorious in science. He has laid his "discovery" before the President of these United

States, and begs that it be speedily placed in the hands of the cholera sufferers and the cholera scared people of the world. The time has come! Cholera, like smallpox, must now bow before the power of science and be disarmed of all its terrors.

The letter to the President, Dr. Leach sends to the TEXAS MEDICAL JOURNAL, accompanied by a courteous request; knowing that we will hasten to give the world the benefit of it in case Benjamin Harrison is too busy to attend to it; knowing, too, our appreciation of a *good thing*. (Here followed the letter. Omitted here.)

In presenting this remarkable document to the physicians of Texas, we hardly know whether to venture a criticism or not. The thing is so palpably absurd, looked at from the standpoint of rational medicine, and in the light of modern sanitary knowledge, that it were useless to combat any assertion therein made. The doctor's premises are false, and, of course, his deductions are wrong. It seems that he does not, in the least, grasp the idea of immunity and infection. The introduction of arsenic into the system can not be compared [as he compares it] to vaccination with an attenuated virus. * * * Who could ever be brought to believe for a moment that because a person has once experienced the effects of arsenic, he would be insusceptible to the effects of a second dose? Or that arsenic, because it produces sometimes symptoms resembling those of cholera, will prevent the action of the cholera poison—a living, deadly microbe. It is too absurd for serious consideration. * * *

There is one thing we admire about this letter; the writer's assurance. He has much to learn yet, but he does not seem to be aware of it. We recommend him to read Frankel's *Bacteriology* and McLaughlin's *Physical Theory of Immunity and Contagion.* He assures us that the "test" is being made at the New York quarantine. The profession will await with anxiety and impatience for the result. He also says the United States Marine Hospital Bureau surgeons have pronounced his "assertions" [sic] impregnable! [That is a pretty good joke on Surgeon General Wyman.] * * *

The cholera scare subsided almost as rapidly as it arose; the disease disappeared. Physicians were at a loss to account for it; but here we have the explanation. The raging lion has retired before this new enemy,—arsenic,—realizing the uselessness of measuring strength with such a remedy, in the hands of such a genius;—even as Beelzebub fled and cowed before the emblem of the cross.

Now, let not the scornful and the envious say that the (alleged) control of yellow fever in New Orleans simultaneously with the advent of this genius, and the saturation of her population with arsenious acid *a la Leach,* was a coincidence, and that the M. H. S. and the powers were just getting in their work. I know better. It was homeopathy and arsenic. Now let Souchon, Kohnke and White go away back and sit down. *Vive la homo!*

### SUMMER HEAT

**does not exhaust the vitality of the invalid fortified by**

### GRAY'S Glycerine TONIC Comp.

**It is the ideal hot weather remedy for the sufferer from chronic organic disease, nervous exhaustion, malnutrition and general debility**

**THE PURDUE FREDERICK CO.,**
**298 Broadway, New York**

The outbreak of yellow fever at Natchez, Mississippi, is a little puzzling, as Natchez has been under a bomb-proof quarantine since the middle of July. Stegomyias must fly high and far in Mississippi.

